# Deep-Learning-Based Mixture Identification for Nuclear Magnetic Resonance Spectroscopy Applied to Plant Flavors

**DOI:** 10.3390/molecules28217380

**Published:** 2023-11-01

**Authors:** Yufei Wang, Weiwei Wei, Wen Du, Jiaxiao Cai, Yuxuan Liao, Hongmei Lu, Bo Kong, Zhimin Zhang

**Affiliations:** 1 College of Chemistry and Chemical Engineering, Central South University, Changsha 410083, China; 222311036@csu.edu.cn (Y.W.); 212311021@csu.edu.cn (Y.L.); hongmeilu@csu.edu.cn (H.L.); 2 Technology Center, China Tobacco Hunan Industrial Co., Ltd., Changsha 410014, China; weiww0216@hngytobacco.com (W.W.); duw0621@hngytobacco.com (W.D.); jassix@foxmail.com (J.C.)

**Keywords:** mixture identification, spatial pyramid pooling, NMR, plant flavors

## Abstract

Nuclear magnetic resonance (NMR) is a crucial technique for analyzing mixtures consisting of small molecules, providing non-destructive, fast, reproducible, and unbiased benefits. However, it is challenging to perform mixture identification because of the offset of chemical shifts and peak overlaps that often exist in mixtures such as plant flavors. Here, we propose a deep-learning-based mixture identification method (DeepMID) that can be used to identify plant flavors (mixtures) in a formulated flavor (mixture consisting of several plant flavors) without the need to know the specific components in the plant flavors. A pseudo-Siamese convolutional neural network (pSCNN) and a spatial pyramid pooling (SPP) layer were used to solve the problems due to their high accuracy and robustness. The DeepMID model is trained, validated, and tested on an augmented data set containing 50,000 pairs of formulated and plant flavors. We demonstrate that DeepMID can achieve excellent prediction results in the augmented test set: ACC = 99.58%, TPR = 99.48%, FPR = 0.32%; and two experimentally obtained data sets: one shows ACC = 97.60%, TPR = 92.81%, FPR = 0.78% and the other shows ACC = 92.31%, TPR = 80.00%, FPR = 0.00%. In conclusion, DeepMID is a reliable method for identifying plant flavors in formulated flavors based on NMR spectroscopy, which can assist researchers in accelerating the design of flavor formulations.

## 1. Introduction

Nuclear magnetic resonance (NMR) is an essential tool for obtaining valuable information about the composition, structure, and dynamics of molecules. It can analyze mixtures composed of small molecules, offering non-destructive, rapid, and accurate advantages [1]. NMR can detect most organic compounds, making it widely applicable in various fields such as chemistry [2], medicine [3], metabolomics [4,5], food [6], and flavors [7]. NMR is considered less sensitive than mass spectrometry [8,9]. However, it has higher repeatability and can serve as a fingerprint technology to compare, differentiate, or classify samples. Extracting meaningful information from complex spectral data of mixtures is an interesting research area. Metabolomics technology based on NMR [10] has been developed to analyze the composition of multiple metabolites in biological fluids, cells, and tissues. These metabolites contain bioactive small molecules that can be widely applied in various industrial fields, ranging from fragrances, flavors, and sweeteners to natural insecticides and pharmaceuticals [11]. There is a wide variety of small molecule metabolites, many of which are produced through plant secondary metabolism pathways [12]. This technology has led to using NMR fingerprinting and profiling to study plant metabolites such as flavors and fragrances [13,14]. The NMR spectra generated from these complex samples should be analyzed using data processing methods [15], even machine learning [16] and deep learning methods [17] in recent years.

Machine learning is a critical component of artificial intelligence [18,19]. It enables machines to learn and extract information and feature patterns from vast amounts of data for further processing [20,21,22,23,24,25]. Recently, machine learning has witnessed significant advancements due to the availability of enhanced computational resources and novel deep learning algorithms [26,27]. These developments have enabled researchers to better deal with the challenges in chemistry, especially analytical chemistry [28], such as near infrared spectroscopy [29], Raman spectroscopy [30,31,32,33], mass spectrometry [34,35,36,37,38,39,40], chromatography [41,42,43,44,45], and ion mobility spectrometry [46,47,48]. Various machine learning methods have also been gradually applied in NMR spectroscopy [49,50], including complex mixture analysis in omics [16,51]. Recent advances in deep learning have attracted attention in various fields of NMR, including fast field homogenization [52], spectrum reconstruction [53], peak picking [54,55], denoising [56], chemical shift prediction [57], functional group recognition [58], protein assignments [59], and mixture component identification [60].

**Figure 1:**
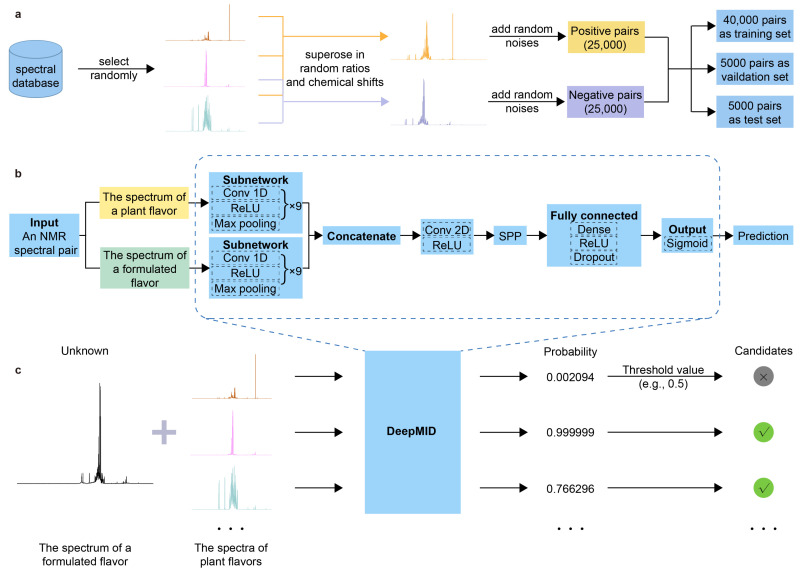
Schematic diagram of DeepMID. (**a**) A spectral database is used for data augmentation, and 50,000 augmented spectral pairs are used for model training, validation, and testing. (**b**) The network architecture of DeepMID. (**c**) DeepMID-based mixture identification.

In this study, a method named DeepMID has been developed for identifying plant flavors in a formulated flavor with NMR spectroscopy inspired by the pseudo-Siamese convolutional neural network (pSCNN) [61] and DeepRaman [62]. The schematic diagram of DeepMID is shown in Figure 1. It mainly consists of three components: data augmentation, pseudo-Siamese convolutional neural network, and model-based mixture identification. As shown in Figure 1a, the NMR spectra from the plant flavor database were augmented to form NMR spectral pairs and further feed into the network. The data augmentation is performed by superimposing several NMR spectra sampled at random ratios from the plant flavor database with random noise to generate negative and positive spectral pairs. Then, the data set is divided into a training set, a validation set, and a test set. Figure 1b shows the network architecture of DeepMID, which takes NMR spectral pairs as inputs, extracts high-level features using convolutional layers, reorganizes these features in the SPP layer, and finally predicts the probability of plant flavors in the formulated flavors by dense layers. Figure 1c shows the mixture identification based on the DeepMID model, which predicts the probability of each plant flavor in a mixture of plant flavors. The possible plant flavors of the formulated flavor can be obtained by filtering the predicted probabilities using a threshold value. We will clarify the principles of each part of this method as clearly as possible in Section 3.

## 2. Results and Discussion

### 2.1. Implementation, Optimization, and Training of DeepMID

The neural networks were implemented in Python (version 3.10.5) and Tensorflow (version 2.5.0-GPU). The NMR spectra were read into Python by nmrglue (version 0.8) [63]. The computing tasks were submitted to the Inspur TS10000 high-performance computing (HPC) cluster of Central South University using the Slurm workload manager (version 20.02.3). This HPC cluster has 1022 central processing unit (CPU) nodes, 10 fat nodes, and 26 graphics processing unit (GPU) nodes. For the training of DeepMID models, it was a GPU node with 2 × Intel(R) Xeon(R) Gold 6248R processors, 2 × Nvidia Tesla V100s, 384G DDR4 memory, and a CentOS 7.5 operating system.

**Figure 2:**
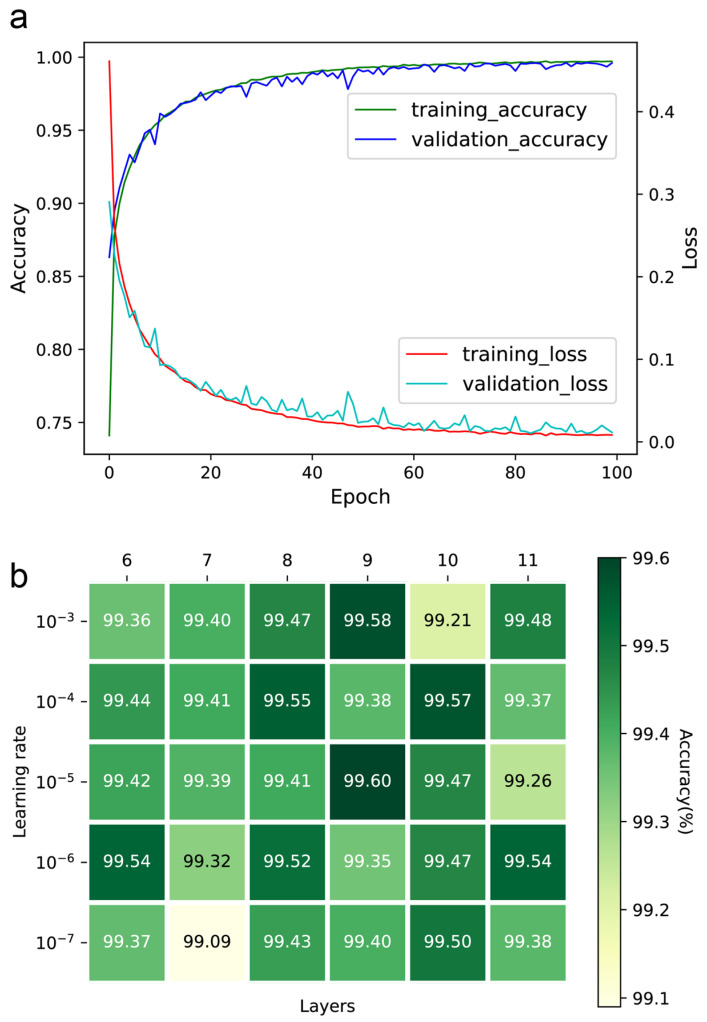
Hyperparameter optimization of the DeepMID model. (**a**) The accuracy curves and loss curves of the training set and validation set. (**b**) The accuracy of different models on the validation set with different hyperparameters.

The number of epochs, learning rate, and convolution layers were optimized to ensure the performance of the DeepMID model. The accuracy and loss curves of the training and validation sets can be found in Figure 2a. Ultimately, the number of epochs was set to 100, as further increases did not significantly contribute to accuracy. Setting the learning rate too low will increase training time and may result in overfitting while setting it too high makes it difficult for the model to converge. The learning rates were optimized in the range from 10^−3^ to 10^−7^, while the number of convolution layers was from 6 to 11. The accuracies of the models are in Figure 2b. It was found that the highest accuracy was achieved with a learning rate of 10^−5^ and 9 convolution layers. Thus, the learning rate was set to 10^−5^, and the number of convolution layers was set to 9.

### 2.2. Evaluation of the DeepMID Model

**Figure 3:**
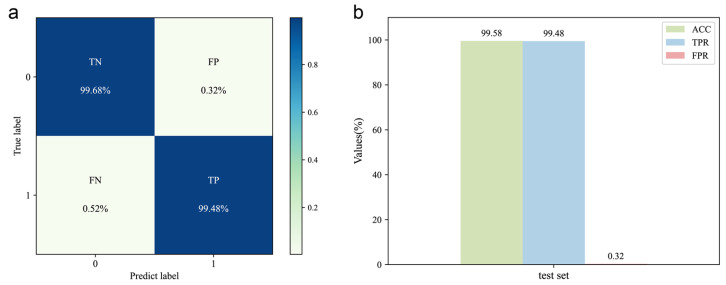
Evaluation of the DeepMID model. (**a**) Confusion matrix of the DeepMID model on the test set. (**b**) Performance evaluation on the test set.

The DeepMID model is trained on the training set (consisting of 40,000 data pairs) obtained by data augmentation. The hyperparameters were optimized by the validation set (consisting of 5000 data pairs). Upon testing the model with the test set, the confusion matrix is shown in Figure 3a, and the corresponding evaluation results are shown in Figure 3b. The results show ACC = 99.58%, TPR = 99.48%, and FPR = 0.32%, indicating high accuracy and generalization capability. Therefore, it can be used for mixture identification of the formulated flavors.

### 2.3. Results of Mixture Identification

**Figure 4:**
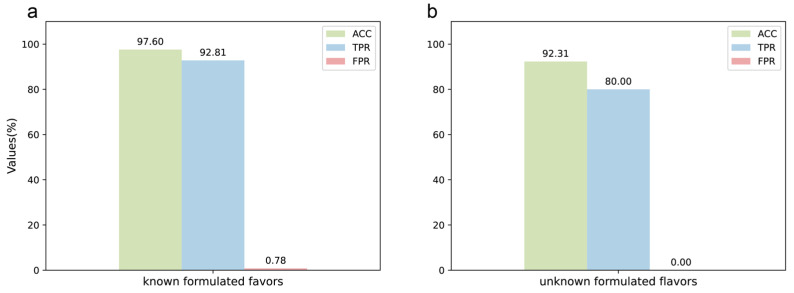
Application of the DeepMID model. (**a**) The result of the known formulated flavors. (**b**) The result of the unknown formulated flavors.

The DeepMID model performed well on the test set of data augmentation, so it was further applied to the known formulated flavor data set. For known formulated flavors, they were made by experimentally blending plant flavors together. The formulations are known, so they can be used to test the performance of the model on real experimental data. The preparation methods and formulations of known formulation flavors are described in Section 4.2 and listed in Table S1. The result is in Figure 4a, showing ACC = 97.60%, TPR = 92.81%, and FPR = 0.78%. The detailed results can be found in Table S2. It can be seen that the model has high accuracy and specificity on the experimental data set and can identify plant flavors from the formulated flavors well without the need to know the specific components in the plant flavors.

### 2.4. Elucidation of Unknown Formulated Flavors

Finally, the model was used to predict candidates of unknown formulated flavors provided by the Technology Center of China Tobacco Hunan Industrial Co., Ltd, Changsha, China. The unknown formulated flavors were used for practical applications, and the formulations of these formulated flavors were unknown before submitting the results. Therefore, the unknown formulated flavors data set could be used to check the performance of the model in practical applications, including ACC, TPR, and FPR. As Figure 4b shows, ACC = 92.31%, TPR = 80.00%, and FPR = 0.00%. The information on the unknown formulated flavors is shown in Table S3. There were two unknown formulated flavors, L1 and L2. L1 was predicted by DeepMID first, and it was found that all the plant flavors predicted were correct against the results sent by the Technology Center of China Tobacco Hunan Industrial Co., Ltd, Changsha, China. The result on L1 could achieve ACC = 100%, TPR = 100%, and FPR = 0.00%. Therefore, DeepMID was further applied to the unknown formulated flavor L2. L2 was a formulated flavor that was used in the actual production application. L1 and L2 were both formulated by five plant flavors. L1 was not diluted with propylene glycol, while L2 was diluted with 50% propylene glycol. The results of the DeepMID model in L2 were ACC = 84.62%, TPR = 60.00%, and FPR = 0.00%. Although the results predicted by the DeepMID model decreased after a large amount of propylene glycol dilution, it still correctly predicted three components, which can assist in the manual formulation elucidation of plant flavors in a formulated flavor to some extent. The detailed results are listed in Table S4.

### 2.5. Stability of the DeepMID Model

**Figure 5:**
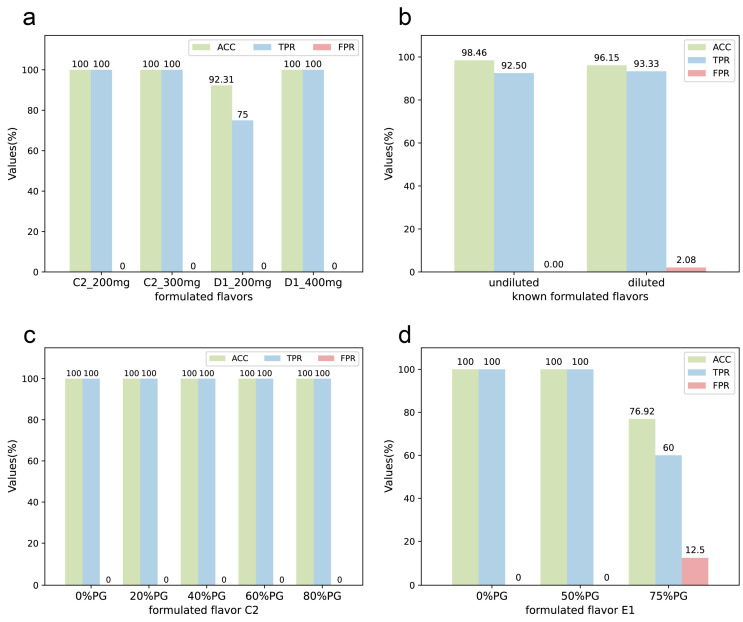
The stability of the DeepMID model. (**a**) For formulated flavor C2, the results on the different qualities of formulated flavors are the same. For D1, better results are obtained with larger qualities. (**b**) The results of undiluted and diluted known formulated flavors can be achieved with little difference. (**c**) The results of the 3-component formulated flavors C1 diluted with different concentrations of PG are the same. (**d**) For the 5-component formulated flavor E1, the results were unaffected when the PG ≤ 50%, but the results decreased when the dilution was greater than 75%.

In order to investigate the performance of the model for formulated flavors with different qualities, we prepared two sets of known formulated flavors with the same formula but different qualities of plant flavors: C2 and C2-300, D1 and D1-400, as shown in Table S5. The total weight of C2 and D1 is 200 mg, while C2-300 is 300 mg and D1-400 is 400 mg. The results are presented in Figure 5a. For the 3-component formulated flavor C2, there was no significant effect of the different qualities on the results. For the 4-component formulated flavor D1, the results became more accurate when the amount of plant flavor in the formulated flavor was increased. However, since most of the experimental plant flavors are viscous and not highly soluble, too much amount used in the preparation of NMR test samples may cause precipitation, and if the solution is too viscous, it may also lead to a decrease in the quality of the NMR spectra, so the total weight of the samples was finally set to use 200 mg. It can be observed that the model was able to provide accurate predictions even when samples of different qualities were used in the preparation process.

In practical applications and production, the formulated flavors are usually diluted with propylene glycol, which may mask characteristic peaks and make it challenging to identify plant flavors in them. Therefore, a gradient experiment with propylene glycol-diluted formulated flavors was conducted. Two sets of known formulated flavors were prepared with different concentrations of PG dilution. C2 was a mixture of three plant flavors; 20%, 40%, 60%, and 80% PG was added to C2 to dilute it. Then, the diluted formulated flavors were named C2-20%, C2-40%, C2-60%, and C2-80%. Similarly, E1 was a known formulated flavor by mixing five plant flavors. After diluting with 50% and 75% PG, the diluted formulated flavors were named E1-50% and E1-75%. The results are shown in Figure 5b, indicating that although propylene glycol dilution can pose some challenges to the identification performance of DeepMID, the accuracy rate remains above 80%. The details of the formulations and the results of mixture identification can be found in Table S1 and Table S2. As shown in Figure 5c,d, the accuracy of the DeepMID model can reach 100% at less than 50% dilution. Hence, dilution has a greater effect on formulated flavors with more components. Though the results decrease as the dilution increases, they are still acceptable, which shows the stability of the DeepMID model in predicting diluted formulated flavors.

### 2.6. Expansion of the Spectral Database

**Figure 6:**
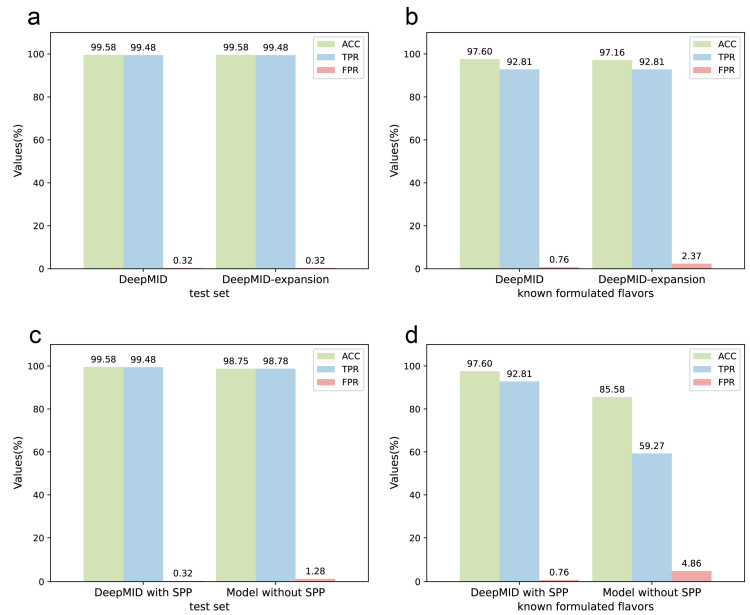
The discussions of the DeepMID model. (**a**) The results of the NMR data sets after expanding the spectral database are the same as before the expansion on the test set. (**b**) The results of the NMR data sets after expanding the spectral database on the known formulated flavors data set show that ACC decreases slightly and FPR increases marginally while TPR does not change compared to before expanding. (**c**) The comparison of the DeepMID model with and without the SPP layer on the test set shows that the SPP layer can improve the results. (**d**) The comparison of the DeepMID model with and without the SPP layer on the known formulated flavors set shows that the results of the model without the SPP layer are much worse than DeepMID with the SPP layer.

Furthermore, 20 plant flavors were added to the database, expanding it to a total of 33 plant flavors. The database initially contained 13 plant flavors, which was expanded to 33. The details of the database before and after the extension are listed in Table S6. Figure 6a,b show the results of DeepMID on the expanded database for mixture identification. The details of the result can be found in Table S7. It can be seen that the results of DeepMID on the test set after expanding the spectral database are the same as before the expansion. For the data set obtained from the experiments, the sensitivity does not change, while the accuracy decreases slightly. The newly added 20 plant flavors have a negligible impact on the results, indicating that the model is reasonably stable, and the later added plant flavors to the database will not deteriorate the prediction performance of the DeepMID model.

### 2.7. Comparison with the Model without the SPP Layer

In Figure 6c,d, the results of DeepMID with and without the SPP layer are compared. The model without the SPP layer works much worse on the experimental data set than the DeepMID model with the SPP layer. While DeepMID can achieve the result that ACC = 97.60%, TPR = 92.81%, and FPR = 0.76% on the known formulated flavors, the model without the SPP layer can only obtain the result that ACC = 85.58%, TPR = 59.27%, and FPR = 4.86%. The detailed results of the model without SPP can be found in Table S8. The SPP can extract spatial feature information of different sizes, which can improve the robustness of the model for the chemical shifts. The addition of the SPP layer allows the DeepMID model to have better generalization ability and prediction performance on experimental NMR spectra of real formulated flavors.

## 3. Methods

### 3.1. Data Set Curation

Many interferences make it challenging to analyze and extract features from the origin of NMR spectra. The following steps are taken to remove these interferences. During NMR experiments, baselines of the spectra may be distorted for many complex reasons. Therefore, adaptive iteratively reweighted penalized least squares (airPLS) [64] is used to correct baselines in NMR spectra. Plant flavors are mixtures of biologically active small molecules extracted from plants. In the extraction and preparation of plant flavors, a large amount of solvent is often added for dilution, which makes it challenging to identify the substance of the plant flavors. In order to solve this problem, the solvent peak is set to zero to enlarge the relative intensity of the characteristic peaks of each plant flavor. Since signal intensities in NMR spectra vary widely, it is necessary to normalize them. Normalization can adjust the intensities of NMR spectra in the same order of magnitude and make them comparable. In addition, normalization can speed up the convergence during gradient descent and increase the speed of finding the optimal solution. Here, the spectra are normalized after zeroing the solvent peaks, which can further amplify the characteristic peaks of plant flavors. Deep learning requires a large amount of data. Therefore, the informative intervals with NMR peaks (0.300–10.700 ppm) are cut out for data augmentation to obtain a data set as large as possible.

**Figure 7:**
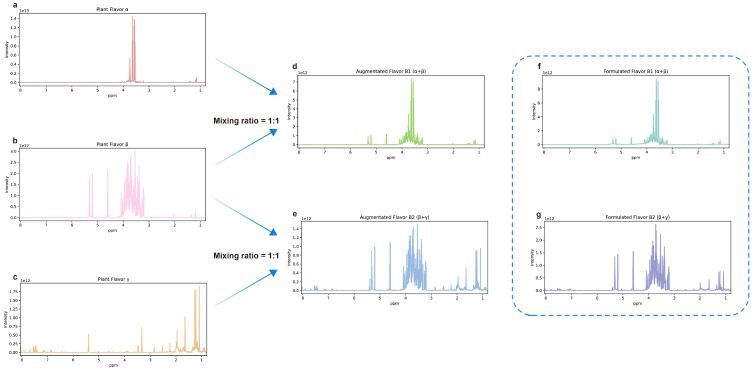
Schematic diagram of NMR spectra and data augmentation. (**a**) The NMR spectrum of plant flavor α. (**b**) The NMR spectrum of plant flavor β. (**c**) The NMR spectrum of plant flavor γ. (**d**) The augmented NMR spectrum of B1 (α + β) obtained by superimposing the spectra of α and β. (**e**) The augmented NMR spectrum of B2 (β + γ) obtained by superimposing the spectra of β and γ. (**f**) The NMR spectrum of formulated flavor B1 (α + β) obtained by experiments. (**g**) The NMR spectrum of formulated flavor B2 (β + γ) was obtained by experiments.

After the preprocessing, a data augmentation method was developed to generate NMR spectral pairs based on the characteristics of NMR spectra. The generated NMR spectral pairs have two different types: positive and negative. Several (1–5) NMR spectra (sampled spectra) are sampled from the plant flavor database and superposed to obtain the NMR spectrum of the formulated flavor. For a positive pair, its plant flavor is a component of its formulated flavor, and the NMR spectrum of its plant flavor is sampled from the sampled spectra. For a negative pair, its plant flavor is not a component of its formulated flavor, and the NMR spectrum of its plant flavor is sampled from the remaining spectra in the database. If the plant flavor is in the formulated flavor, the corresponding label of the NMR spectral pair is 1. Otherwise, it is 0. For example, Figure 7a shows the NMR spectrum of hops extract, named plant flavor α. Figure 7b shows the NMR spectrum of fig extract, named plant flavor β. Figure 7c shows the NMR spectrum of Roman chamomile extract, named plant flavor γ. The spectra of hops extract (α) and fig extract (β) were multiplied by a ratio of 0.5 and then superimposed to obtain the augmented NMR spectra of the formulated flavor B1 (α + β), as shown in Figure 7d. In the same way, the spectra of fig extract (β) and Roman chamomile extract (γ) were superimposed to obtain the augmented NMR spectra of the formulated flavor B2 (β + γ). For plant flavor α, the spectra pair that it combines with B1 (α + β) is a positive pair, while the spectra pair that it forms with B2 (β + γ) is a negative pair. For plant flavor β, both formulated flavors B1 and B2 combined with it are positive pairs. Formulated flavors B1 and B2 were formulated according to the above formula, and the NMR spectra are shown in Figure 7f,g. It can be observed that the augmented data can represent the experimental data to some extent. Generally speaking, the solvent peaks in the experimental data tend to be higher, while the characteristic peaks of plant flavors are lower than them. Because of the noise and chemical shift drift in real NMR spectra, random Gaussian noise and chemical shift drift (−3 × 10^−4^, 3 × 10^−4^ ppm) are injected into the plant flavors NMR spectra for each spectral pair separately. Due to the varied concentration, each NMR spectrum is multiplied by a random scale factor (0.2–1.0) when generating the mixture NMR spectrum. With the proposed data augmentation method and the plant flavor database, 50,000 NMR spectral pairs were generated, with 50% each of positive and negative samples. These spectral pairs were randomly divided into the training, validation, and test sets in the ratio of 8:1:1.

### 3.2. Pseudo-Siamese Neural Network

The Siamese network (SNN) [65] consists of two identical subnetworks that share weights, taking in two input samples and producing their corresponding embeddings. These embeddings are then fed into a similarity function to compute the similarity or dissimilarity between the inputs. However, spectra of plant flavor and formulated flavor in an NMR spectral pair differ significantly. The pseudo-Siamese neural network (pSNN) is a variant of the SNN architecture that aims to perform similarity-based tasks. It contains two convolution neural networks with the same architecture, which do not share their weights. pSNN used here has two similar subnetworks to extract feature vectors from the spectral pairs. Then, the features are concatenated and fed to the next layer for comparison. Compared to the SNN architecture, pSNN is a more suitable architecture for the mixture identification task.

### 3.3. Spatial Pyramid Pooling

**Figure 8:**
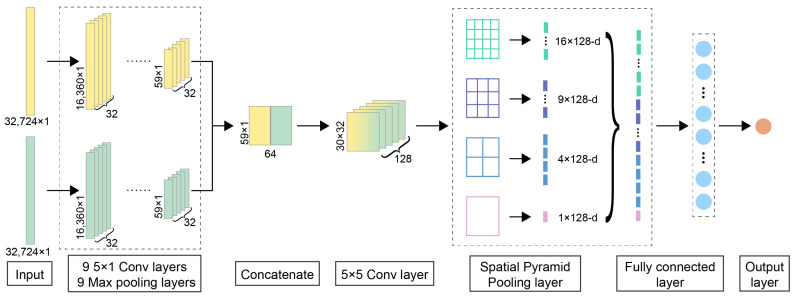
The detailed neural network architecture of DeepMID. DeepMID takes NMR spectral pairs as inputs, each comprising two NMR spectra: a plant flavor spectrum and a formulated flavor spectrum. The high-level feature maps from the spectral pairs were extracted by two subnetworks with the same architecture and then flattened and concatenated. Further extract features are obtained by a two-dimensional convolutional layer. An SPP layer pools these feature maps to produce a fixed-length feature, which then passes through a dense layer for comparison. The final output is obtained from an output layer with one unit.

The spatial pyramid pooling [66] layer can pool varied feature maps and produce fixed-length outputs by dividing the feature maps into regions and pooling features in each region. The basic idea behind SPP is to divide the input feature maps into several grids or levels, with each level capturing features at a different scale. Feature pooling is performed within each grid or level to generate a fixed-length representation. These representations are then concatenated to form the final feature representation. SPP allows the network to capture both global information and local details, regardless of the size or aspect ratio of the input feature maps. As shown in Figure 8, for the two-dimensional feature map obtained after the two-dimensional convolution, the feature maps are pooled in the SPP layer with (4 × 4, 3 × 3, 2 × 2, 1 × 1) multi-level windows to obtain 4 × 4 = 16 feature vectors of 1 × 128, 3 × 3 = 9 feature vectors of 1 × 128, 2 × 2 = 4 feature vectors of 1 × 128, and one 1 × 128 feature vectors that are pooled directly to the entire feature map are stitched together to obtain a (16 + 9 + 4 + 1) × 128 = 30 × 128 feature vector. It is flattened into a one-dimensional 3840 × 1 feature vector and fed into the following fully connected layer. Multi-level windows are robust to object deformation compared to a conventional moving window pool with only a single window size. Thus, SPP can improve scale invariance, avoid distortions caused by traditional moving windows, and reduce overfitting with multi-scale features extracted to a fixed size to be fed into the fully connected layer.

### 3.4. Detailed Network Architecture

The detailed network architecture of DeepMID is shown in Figure 8. The inputs of DeepMID are NMR spectral pairs, and each spectral pair consists of two NMR spectra. One is the NMR spectrum of a plant flavor, and the other is the NMR spectrum of a formulated flavor. Two subnetworks with the same architecture (9 one-dimensional convolutional blocks) are chosen to extract the high-level feature maps from two NMR spectra in each spectral pair. Each convolutional block of subnetworks has a convolutional layer followed by a rectified linear unit (ReLU) activation layer and a max pooling layer. The weight and bias of convolutional kernels were initialized by the He normal initializer [67]. The number of convolutional kernels is 32, and the kernel size is 5 × 1. The feature maps extracted by the subnetworks are flattened and concatenated. A two-dimensional convolutional layer is used for further feature extraction; the number of kernels is 128, and the kernel size is 5 × 5. Then, the feature maps are pooled by an SPP layer to generate a feature with a fixed length, and the SPP layer is a 4-level pyramid with 30 bins (4 × 4, 3 × 3, 2 × 2, 1 × 1). The fixed-length feature is fed into a dense layer for comparison. There are 100 hidden units in the dense layer. Its activation function is also the ReLU function, and a dropout layer with a dropout rate equaling 0.2 is used to reduce the risk of overfitting. Finally, the output layer with one unit forms the final output, and its activation function is the Sigmoid function. The loss function is the binary cross-entropy, which applies to binary classification. The Adam [68] optimizer is chosen as the optimizer.

### 3.5. Mixture Identification

After the DeepMID model is established, it can elucidate the formulated flavors. A formulated flavor is composed of multiple plant flavors according to a formula. Plant flavors are mixtures. Therefore, a formulated flavor is a mixture of mixtures. The identification of plant flavors in a formulated flavor is the identification of mixtures in a mixture of mixtures. Therefore, this process has been termed as **mixture identification**. The detailed identification process is as follows. The NMR spectrum of a formulated flavor is denoted as a vector **s**. All the NMR spectra of plant flavors in a spectral library can be represented as a matrix **D** (*N* × *P*), *N* is the number of plant flavors in the library, and P is the number of data points in each NMR spectrum. Combining the NMR spectrum (**s**) of a formulated flavor with each NMR spectrum (D_i_) in the database yields *N* spectral pairs (s, D_1_), …, (s, D_N_). Each spectral pair is predicted by the DeepMID model to obtain the probability of a plant flavor in the formulated flavor. A threshold value (e.g., 0.5) can be set for this probability to control the number of false positives. If the probability of a plant flavor in the formulated flavor is larger than the threshold, it can be identified as a candidate. After completing the prediction and filtering of *N* spectral pairs, a list of candidates for the presence of plant flavors in the formulated flavor is obtained.

### 3.6. Evaluation Metrics

Accuracy rate (ACC), true positive rate (TPR, sensitivity), and false positive rate (FPR, 1-specificity) were used to evaluate the performance of the models in this study. The formulas for ACC, TPR, and FPR are as follows:

(1) $$\mathrm{ACC}=\frac{\mathrm{TP}+\mathrm{TN}}{\mathrm{TP}+\mathrm{TN}+\mathrm{FP}+\mathrm{FN}}$$

(2) $$\mathrm{TPR}=\frac{\mathrm{TP}}{\mathrm{TP}+\mathrm{FN}}$$

(3) $$\mathrm{FPR}=\frac{\mathrm{FP}}{\mathrm{TN}+\mathrm{FP}}$$

Here, TP, FP, TN, and FN stand for true positive, false positive, true negative, and false negative, respectively. In binary classification, samples are marked as positive or negative. If the plant flavor is in the formulated flavor, it is marked as positive, and vice versa for negative. If the predicted and actual values are both positive, we consider it a true positive (TP). If they are both negative, it is a true negative (TN). However, if the predicted value is positive and the actual value is negative, we classify it as a false positive (FP). On the other hand, if the predicted value is negative but the actual value is positive, we classify it as a false negative (FN). For example, if the model predicts that the probability of a plant flavor being in a formulated flavor is >0.5, the predicted value is positive, and the plant flavor is actually presented in the formulated flavor, which means that the true value is positive. Then, it is true positive.

## 4. Experiments

### 4.1. Plant Flavors

The plant flavors were provided by third-party personnel in the Technology Center of China Tobacco Hunan Industrial Co., Ltd., Changsha, China. These plant flavors are often used to prepare formulated flavors. Most of these plant flavors were mixtures of metabolites extracted from plants containing bioactive small molecules, and their compositions were complex and unidentified. The samples of plant flavors were produced by dissolving 200 mg of the natural plant flavor into a mixture of 0.6 mL CH_3_OD and 0.6 mL Phosphate Buffer (PB), and the detail of PB is shown in Figure S1. After being vortexed at 500 rpm for 1 min, ultrasonicated for 15 min, and centrifuged at 13,000 rpm for 10 min at 15 °C, the supernatant was taken at 550 μL for NMR measurement. Sample measurements were performed at 25 °C on a Bruker AVANCE NEO 600 MHz NMR spectrometer with the Prodigy cryoprobe (Bruker Scientific Instruments, Fällanden, Switzerland), and the cryoprobe was operated at the temperature of liquid nitrogen. The measurement frequency was ^1^H NMR 600.12 MHz, and the number of scans was 128. The pulse program was chosen as noesypprld to suppress the water peak; TSP was the internal standard; MeOD was used for the NMR field locking. The information on each flavor is listed in Table S9, and the information on the reagents is listed in Table S10.

### 4.2. Known Formulated Flavors

In addition, 16 mixtures with known plant flavors were prepared and measured through real experiments as another data set to test the DeepMID model. These blends, formulated from plant flavors (mixtures), were called known formulated flavors. These formulated flavors were numbered according to their composition. For instance, the formulated flavors consist of two plant flavors numbered B1 and B2 and three plant flavors numbered C1 and C2. The formulated flavors with more components are named similarly. The specific formulations are listed in Table S3. They were dissolved into a solution of 0.6 mL CH_3_OD and 0.6 mL PB. The following steps and the NMR measurement conditions are the same as plant flavors.

### 4.3. Unknown Formulated Flavors

Two unknown formulated flavors were provided by third-party personnel in the Technology Center of China Tobacco Hunan Industrial Co., Ltd., Changsha, China. We were told the plant flavors of the formulated flavors only after submitting the predicted result. The samples were prepared by dissolving 200 mg of the unknown formulated flavors into a mixture of 0.6 mL CH_3_OD and 0.6 mL PB and then were processed in the same steps as plant flavors. In addition, the NMR measurement experimental conditions were set as the plant flavors.

## 5. Conclusions

In this research, DeepMID was proposed to identify plant flavors in formulated flavors using a pseudo-Siamese neural network and ^1^H NMR spectroscopy.

First, we continuously adjusted the experimental protocol for our plant flavors to determine the best method and obtained NMR spectra. Compared to mass spectrometry, NMR has a lower sensitivity, making it difficult to accurately identify plant flavors with complex compositions and low concentrations in formulated flavors. To address this issue, a 600 MHz NMR spectrometer was chosen instead of a 400 MHz NMR spectrometer, and the Prodigy cryoprobe operated at liquid nitrogen temperature was used. Additionally, the number of scans was increased, and the pulse program was chosen as the *noesypprld* to suppress the water peak. Furthermore, we conducted experimental comparisons to determine the most suitable deuterium solvent for dissolving more plant flavors.

Next, after obtaining high-quality NMR spectra, we can input them into the network for training. The probabilities of the plant flavors in a formulated flavor can be obtained by feeding the spectral pairs of plant flavors and the formulated flavors into DeepMID. Then, 50,000 pairs of spectra were generated by superposing the NMR spectra in the plant flavor database. They were randomly divided into the training set, validation set, and test set in the ratio of 8:1:1 for training, validation, and testing the DeepMID model. The performance of the model was tested by the test set and experimental data sets in terms of ACC, TPR, and FPR.

Finally, DeepMID achieved good results in the augmented test set: ACC = 99.58%, TPR = 99.48%, and FPR = 0.32%, and the results of known formulated flavors show ACC = 97.60%, TPR = 92.81%, and FPR = 0.76%. The DeepMID model can also achieve acceptable performance with mixtures with different qualities, PG dilution mixtures, and expansion of the spectral database. The model identified plant flavors in unknown formulated flavors and achieved good results in mixture identification: ACC = 92.31%, TPR = 80.00%, and FPR = 0.00%.

In summary, DeepMID is a valuable method for identifying plant flavors in formulated flavors based on NMR spectroscopy, which can effectively identify plant flavors containing small molecules and can be extended to more application scenarios for NMR spectroscopy.

## Data Availability

The source code, model, spectra, manual, and tutorial are available at https://github.com/yfWang01/DeepMID and https://doi.org/10.5281/zenodo.10033745 (accessed on 31 October 2023).

## Supplementary Materials

The following supporting information can be downloaded at: https://www.mdpi.com/article/10.3390/molecules28217380/s1, Figure S1. The preparation method of phosphate buffer; Table S1. The information of the known formulated flavors; Table S2. The detailed results of the known formulated flavors data set; Table S3. The information of the unknown formulated flavors; Table S4. The detailed results of the unknown formulated flavors data set; Table S5. The information of the formulated flavors with different qualities; Table S6. The plant flavors of the database before and after the extension; Table S7. The detailed results of the DeepMID model after extension on the known formulated flavors data set; Table S8. The detailed results of the DeepMID model without SPP layer on the known formulated flavors data set; Table S9. The information about plant flavors; Table S10. Detailed information of the reagents.
