# Supplementary material for: Deep-Learning-Based Mixture Identification for Nuclear Magnetic Resonance Spectroscopy Applied to Plant Flavors
Source: Molecules. 2023 Nov 1;28(21):7380. doi: 10.3390/molecules28217380 (PMC10648966; doi:10.3390/molecules28217380)
Supplement: Supplementary file 1 [file molecules-28-07380-s001.zip › molecules-2661497-supplementary.pdf]

molecules

Article

Deep Learning-based Mixture Identification for Nuclear Magnetic Resonance Spectroscopy Applied to Plant Flavors

Yufei Wang[1],Weiwei Wei[2], Wen Du[2], Jiaxiao Cai[2], Yuxuan Liao[1], Hongmei Lu[1], Bo Kong[2,*] and Zhimin Zhang[1,*]

[1] College of Chemistry and Chemical Engineering, Central South University, Changsha 410083, China 222311036@csu.edu.cn (Y.W.); 212311021@csu.edu.cn (Y.L.); hongmeilu@csu.edu.cn (H.L.)
[2] Technology Center, China Tobacco Hunan Industrial Co., LTD, Changsha 410014, China weiww0216@hngytobacco.com (W.W.); duw0621@hngytobacco.com (W.D.); jassix@ foxmail.com (J.C.)
* Correspondence: zmzhang@csu.edu.cn (Z.Z.); kongb0706@hngytobacco.com (B.K.)

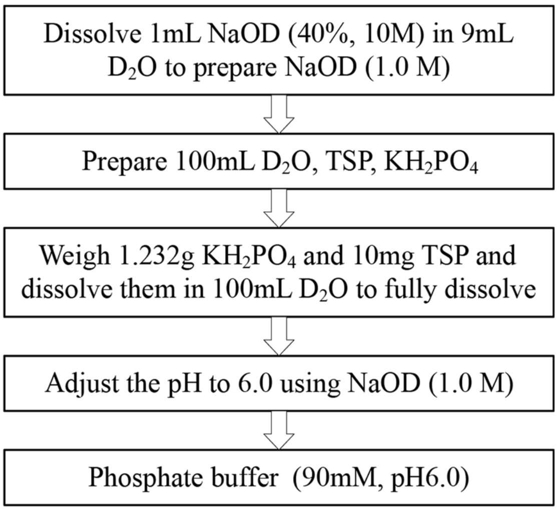

Figure S1. The preparation method of phosphate buffer

Table S1. The information of the known formulated flavors.

Formulated flavors	Formulation	Ratio
B1	Hops Extraction	0.5
	Fig Extraction	0.5
B2	Chamomile Extraction-A	0.5
	Fig Extraction	0.5
C1	Roman Chamomile Extraction-A	0.33
	Tobacco Maillard Reactants	0.33
	Valerian Root Extraction	0.33
C2	Carob Extraction	0.33
	Raisin Extraction	0.33
	Valerian Root Extraction	0.33
C2-300	Carob Extraction	0.33
	Raisin Extraction	0.33
	Valerian Root Extraction	0.33
C2-20%	Carob Extraction	0.26
	Raisin Extraction	0.26
	Valerian Root Extraction	0.26
	Propylene Glycol	0.2
C2-40%	Carob Extraction	0.2
	Raisin Extraction	0.2
	Valerian Root Extraction	0.2
	Propylene Glycol	0.4
C2-60%	Carob Extraction	0.13
	Raisin Extraction	0.13
	Valerian Root Extraction	0.13
	Propylene Glycol	0.6
C2-80%	Carob Extraction	0.06
	Raisin Extraction	0.06
	Valerian Root Extraction	0.06
	Propylene Glycol	0.8

Table S1. Cont.

Formulated flavors	Formulation	Ratio
C3	Alfalfa Extraction	0.33
	Plum Extraction	0.33
	Chicory Extraction	0.33
D1	Raisin Extraction	0.25
	Alfalfa Extraction	0.25
	Fig Extraction	0.25
	Hops Extraction	0.25
D1-400	Raisin Extraction	0.25
	Alfalfa Extraction	0.25
	Fig Extraction	0.25
	Hops Extraction	0.25
D2	Tobacco Maillard Reactants	0.25
	Carob Extraction	0.25
	Valerian Root Extraction	0.25
	Plum Extraction	0.25
E1	Carob Extraction	0.2
	Chicory Extraction	0.2
	Alfalfa Extraction	0.2
	Plum Extraction	0.2
	Roman Chamomile Extraction-A	0.2
E1-50%	Carob Extraction	0.1
	Chicory Extraction	0.1
	Alfalfa Extraction	0.1
	Plum Extraction	0.1
	Roman Chamomile Extraction-A	0.1
	Propylene Glycol	0.5
E1-75%	Carob Extraction	0.05
	Chicory Extraction	0.05
	Alfalfa Extraction	0.05
	Plum Extraction	0.05
	Roman Chamomile Extraction-A	0.05
	Propylene Glycol	0.75

Table S2. The detailed results of the known formulated flavors dataset.

No.	Formulation	Prediction results	ACC(%)	TPR(%)	FPR(%)
B1	Hops Extraction<br>Fig Extraction	Hops Extraction	92.31	50.00	0.00
B2	Chamomile Extraction-A<br>Fig Extraction	Chamomile Extraction-A<br>Fig Extraction	100.0	100.0	0.00
C1	Roman Chamomile Extraction-A<br>Tobacco Maillard Reactants<br>Valerian Root Extraction	Roman Chamomile Extraction-A<br>Tobacco Maillard Reactants<br>Valerian Root Extraction	100.0	100.0	0.00
C2	Carob Extraction<br>Raisin Extraction<br>Valerian Root Extraction	Carob Extraction<br>Raisin Extraction<br>Valerian Root Extraction	100.0	100.0	0.00
C2-300	Carob Extraction<br>Raisin Extraction<br>Valerian Root Extraction	Carob Extraction<br>Raisin Extraction<br>Valerian Root Extraction	100.0	100.0	0.00
C2-20%	Carob Extraction<br>Raisin Extraction<br>Valerian Root Extraction	Carob Extraction<br>Raisin Extraction<br>Valerian Root Extraction	100.0	100.0	0.00
C2-40%	Carob Extraction<br>Raisin Extraction<br>Valerian Root Extraction	Carob Extraction<br>Raisin Extraction<br>Valerian Root Extraction	100.0	100.0	0.00
C2-60%	Carob Extraction<br>Raisin Extraction<br>Valerian Root Extraction	Carob Extraction<br>Raisin Extraction<br>Valerian Root Extraction	100.0	100.0	0.00
C2-80%	Carob Extraction<br>Raisin Extraction<br>Valerian Root Extraction	Carob Extraction<br>Raisin Extraction<br>Valerian Root Extraction	100.0	100.0	0.00
C3	Alfalfa Extraction<br>Plum Extraction<br>Chicory Extraction	Alfalfa Extraction<br>Plum Extraction<br>Chicory Extraction	100.0	100.0	0.00
D1	Raisin Extraction<br>Alfalfa Extraction<br>Fig Extraction<br>Hops Extraction	Raisin Extraction<br>Fig Extraction<br>Hops Extraction	92.31	75.00	0.00

Table S2. Cont.

No.	Formulation	Prediction results	ACC(%)	TPR(%)	FPR(%)
D1-400	Raisin Extraction<br>Alfalfa Extraction<br>Fig Extraction<br>Hops Extraction	Raisin Extraction<br>Alfalfa Extraction<br>Fig Extraction<br>Hops Extraction	100.0	100.0	0.00
D2	Tobacco Maillard Reactants<br>Carob Extraction<br>Valerian Root Extraction<br>Plum Extraction	Tobacco Maillard Reactants<br>Carob Extraction<br>Valerian Root Extraction<br>Plum Extraction	100.0	100.0	0.00
E1	Carob Extraction<br>Chicory Extraction<br>Alfalfa Extraction<br>Plum Extraction<br>Roman Chamomile Extraction-A	Carob Extraction<br>Chicory Extraction<br>Alfalfa Extraction<br>Plum Extraction<br>Roman Chamomile Extraction-A	100.0	100.0	0.00
E1-50%	Carob Extraction<br>Chicory Extraction<br>Alfalfa Extraction<br>Plum Extraction<br>Roman Chamomile Extraction-A	Carob Extraction<br>Chicory Extraction<br>Alfalfa Extraction<br>Plum Extraction<br>Roman Chamomile Extraction-A	100.0	100.0	0.00
E1-75%	Carob Extraction<br>Chicory Extraction<br>Alfalfa Extraction<br>Plum Extraction<br>Roman Chamomile Extraction-A	Carob Extraction<br>Plum Extraction<br>Roman Chamomile Extraction-A<br>Raisin Extraction	76.92	60.00	12.50

Table S3. The information of the unknown formulated flavors.

Formulated flavors	Formulation*	Ratio
L1	Hops Extraction	0.2
	Raisin Extraction	0.2
	Alfalfa Extraction	0.2
	Fig Extraction	0.2
	Plum Extraction	0.2
L2	Hops Extraction	0.1
	Raisin Extraction	0.1
	Alfalfa Extraction	0.1
	Fig Extraction	0.1
	Plum Extraction	0.1
	Propylene Glycol	0.5

* This information was not known at the time of our analysis with the model, and we were informed of the corresponding components after submitting the result.

Table S4. The detailed results of the unknown formulated flavors dataset.

No.	Formulation	Prediction results	ACC(%)	TPR(%)	FPR(%)
L1	Hops Extraction<br>Raisin Extraction<br>Alfalfa Extraction<br>Fig Extraction<br>Plum Extraction	Hops Extraction<br>Raisin Extraction<br>Alfalfa Extraction<br>Fig Extraction<br>Plum Extraction	100.00%	100.00%	0.00%
L2	Hops Extraction<br>Raisin Extraction<br>Alfalfa Extraction<br>Fig Extraction<br>Plum Extraction	Hops Extraction<br>Alfalfa Extraction<br>Plum Extraction	84.62%	60.00%	0.00%

Table S5. The information of the formulated flavors with different qualities.

Formulated flavors	Formulation	Ratio	Weight(mg)
C2	Carob Extraction	0.33	66.7
	Raisin Extraction	0.33	66.7
	Valerian Root Extraction	0.33	66.7
C2-300	Carob Extraction	0.33	100.0
	Raisin Extraction	0.33	100.0
	Valerian Root Extraction	0.33	100.0
D1	Raisin Extraction	0.25	50.0
	Alfalfa Extraction	0.25	50.0
	Fig Extraction	0.25	50.0
	Hops Extraction	0.25	50.0
D1-400	Raisin Extraction	0.25	100.0
	Alfalfa Extraction	0.25	100.0
	Fig Extraction	0.25	100.0
	Hops Extraction	0.25	100.0

Table S6. The plant flavors of the database before and after the extension.

Before extension	After extension
Galbanum Extraction	Benzoin Ointment
Carob Extraction	Peruvian Infusion
Chicory Extraction	Tobacco Burley Extraction
Roman Chamomile Extraction-A	Brazil Tobacco Extraction
Roman Chamomile Extraction-B	Maple Maple Extraction
Alfalfa Extraction	Galbanum Extraction
Plum Extraction	Fenugreek Extraction
Hops Extraction	Jujube Extraction
Raisin Extraction	Zimbabwe Tobacco Extraction
Fig Extraction	Carob Extraction
Valerian Root Extraction	Chicory Extraction
Tobacco Maillard Reactants	Coffee Extraction
Yunnan Tobacco Extraction	Cocoa Extraction
	Labdanum Extraction
	Green Tea Extraction
	Roman Chamomile Extraction-A
	Roman Chamomile Extraction-B
	Virginia Tobacco Extraction
	Alfalfa Extraction
	Malt Infusion
	Plum Extraction
	Hops Extraction
	Raisin Extraction
	Seabuckthorn Extraction
	Tamarind Extraction
	Aglain Odorate Concrete
	Turkish Oriental Tobacco Extraction
	Tulip Concrete
	Fig Extraction
	Smoked Plum Extraction
	Valerian Root Extraction
	Tobacco Maillard Reactants
	Yunnan Tobacco Extraction

Table S7. The detailed results of the DeepMID model after extension on the known formulated flavors dataset.

No.	Formulation	Prediction results	ACC(%)	TPR(%)	FPR(%)
B1	Hops Extraction<br>Fig Extraction	Hops Extraction<br>Coffee Extraction	93.94	50.00	3.23
B2	Chamomile Extraction-A<br>Fig Extraction	Chamomile Extraction-A<br>Fig Extraction	100.0	100.0	0.00
C1	Roman Chamomile Extraction-A<br>Tobacco Maillard Reactants<br>Valerian Root Extraction	Roman Chamomile Extraction-A<br>Tobacco Maillard Reactants<br>Valerian Root Extraction	100.0	100.0	0.00
C2	Carob Extraction<br>Raisin Extraction<br>Valerian Root Extraction	Carob Extraction<br>Raisin Extraction<br>Valerian Root Extraction<br>Jujube Extraction	96.97	100.0	3.33
C2-300	Carob Extraction<br>Raisin Extraction<br>Valerian Root Extraction	Carob Extraction<br>Raisin Extraction<br>Valerian Root Extraction	100.0	100.0	0.00
C2-20%	Carob Extraction<br>Raisin Extraction<br>Valerian Root Extraction	Carob Extraction<br>Raisin Extraction<br>Valerian Root Extraction	100.0	100.0	0.00
C2-40%	Carob Extraction<br>Raisin Extraction<br>Valerian Root Extraction	Carob Extraction<br>Raisin Extraction<br>Valerian Root Extraction	100.0	100.0	0.00
C2-60%	Carob Extraction<br>Raisin Extraction<br>Valerian Root Extraction	Carob Extraction<br>Raisin Extraction<br>Valerian Root Extraction<br>Jujube Extraction	96.97	100.0	3.33
C2-80%	Carob Extraction<br>Raisin Extraction<br>Valerian Root Extraction	Carob Extraction<br>Raisin Extraction<br>Valerian Root Extraction	100.0	100.0	0.00

Table S7. Cont.

No.	Formulation	Prediction results	ACC(%)	TPR(%)	FPR(%)
C3	Alfalfa Extraction<br>Plum Extraction<br>Chicory Extraction	Alfalfa Extraction<br>Plum Extraction<br>Chicory Extraction<br>Tamarind Extraction	96.97	100.0	3.33
D1	Raisin Extraction<br>Alfalfa Extraction<br>Fig Extraction<br>Hops Extraction	Raisin Extraction<br>Fig Extraction<br>Hops Extraction<br>Coffee Extraction	93.94	75.00	3.45
D1-400	Raisin Extraction<br>Alfalfa Extraction<br>Fig Extraction<br>Hops Extraction	Raisin Extraction<br>Alfalfa Extraction<br>Fig Extraction<br>Hops Extraction<br>Coffee Extraction	96.97	100.0	3.45
D2	Tobacco Maillard Reactants<br>Carob Extraction<br>Valerian Root Extraction<br>Plum Extraction	Tobacco Maillard Reactants<br>Carob Extraction<br>Valerian Root Extraction<br>Plum Extraction<br>Tamarind Extraction	96.97	100.0	3.45
E1	Carob Extraction<br>Chicory Extraction<br>Alfalfa Extraction<br>Plum Extraction<br>Roman Chamomile Extraction-A	Carob Extraction<br>Chicory Extraction<br>Alfalfa Extraction<br>Plum Extraction<br>Roman Chamomile Extraction-A<br>Tamarind Extraction<br>Jujube Extraction	93.94	100.0	7.14
E1-50%	Carob Extraction<br>Chicory Extraction<br>Alfalfa Extraction<br>Plum Extraction<br>Roman Chamomile Extraction-A	Carob Extraction<br>Chicory Extraction<br>Alfalfa Extraction<br>Plum Extraction<br>Roman Chamomile Extraction-A<br>Jujube Extraction	96.97	100.0	3.57

Table S7. Cont.

No.	Formulation	Prediction results	ACC(%)	TPR(%)	FPR(%)
E1-75%	Carob Extraction<br>Chicory Extraction<br>Alfalfa Extraction<br>Plum Extraction<br>Roman Chamomile Extraction-A	Carob Extraction<br>Plum Extraction<br>Roman Chamomile Extraction-A<br>Raisin Extraction	90.91	60.00	3.57

Table S2. The detailed results of the DeepMID model without SPP layer on the known formulated flavors dataset.

No.	TN	TP	FN	FP	ACC(%)	TPR(%)	FPR(%)
Test set	4936	4939	61	64	98.75	98.78	1.28
B1	10	1	1	1	100.0	100.0	0.00
B2	11	2	0	0	84.62	50.00	9.09
C1	9	3	0	1	92.31	100.0	10.00
C2	10	2	1	0	92.31	66.67	0.00
C2-300	10	2	1	0	92.31	66.67	0.00
C2-20%	10	2	1	0	69.23	0.00	10.00
C2-40%	10	2	1	0	76.92	50.00	11.11
C2-60%	10	1	2	0	92.31	75.00	0.00
C2-80%	10	1	2	0	100.0	100.0	0.00
C3	9	0	3	1	92.31	66.67	0.00
D1	8	2	2	1	92.31	66.67	0.00
D1-400	9	3	1	0	84.62	33.33	0.00
D2	9	4	0	0	84.62	33.33	0.00
E1	7	4	1	1	84.62	80.00	12.50
E1-50%	7	2	3	1	69.23	40.00	12.50
E1-75%	7	1	4	1	61.54	20.00	12.50

Table S9. The information about plant flavors.

Name	Vendor
Benzoin Ointment	Shanghai Qiyuan Flavor & Fragrance Co. Ltd., Shanghai, China
Peruvian Infusion	Hunan Zhufeng Technology Co., Ltd. Changde, China
Tobacco Burley Extraction	Shanghai Qiyuan Flavor & Fragrance Co. Ltd., Shanghai, China
Brazil Tobacco Extraction	Shanghai Qiyuan Flavor & Fragrance Co. Ltd., Shanghai, China
Maple Extraction	Hunan Zhufeng Technology Co., Ltd. Changde, China
Galbanum Extraction	Guangzhou Huafang Tobacco Flavor Co., Ltd., Guangzhou, China
Fenugreek Extraction	Hunan Shafu Trading Co., Changsha, China
Jujube Extraction	Guangdong Bosch Flavor & Fragrance Co., Ltd., Guangzhou, China
Zimbabwe Tobacco Extraction	Hunan Shafu Trading Co., Changsha, China
Carob Extraction	Guangzhou Huafang Tobacco Flavor Co., Ltd., Guangzhou, China
Chicory Extraction	Guangzhou Huafang Tobacco Flavor Co., Ltd., Guangzhou, China
Coffee Extraction	Hangzhou Jiemin Flavor & Fragrance Co., Ltd., Hangzhou, China
Cocoa Extraction	Shanghai Qiyuan Flavor & Fragrance Co. Ltd., Shanghai, China
Labdanum Extraction	Guangzhou Huafang Tobacco Flavor Co., Ltd., Guangzhou, China
Green Tea Extraction	Guangzhou Huafang Tobacco Flavor Co., Ltd., Guangzhou, China
Roman Chamomile Extraction-A	Guangzhou Huafang Tobacco Flavor Co., Ltd., Guangzhou, China
Roman Chamomile Extraction-B	Zhuhai Guanglong Flavor Co., Ltd., Zhuhai, China
Virginia Tobacco Extraction	Shanghai Qiyuan Flavor & Fragrance Co. Ltd., Shanghai, China
Alfalfa Extraction	Guangzhou Huafang Tobacco Flavor Co., Ltd., Guangzhou, China
Malt Infusion	Hunan Zhufeng Technology Co., Ltd. Changde, China
Plum Extraction	Guangzhou Huafang Tobacco Flavor Co., Ltd., Guangzhou, China
Hops Extraction	Guangzhou Huafang Tobacco Flavor Co., Ltd., Guangzhou, China
Raisin Extraction	Guangzhou Huafang Tobacco Flavor Co., Ltd., Guangzhou, China
Seabuckthorn Extraction	Guangdong Bosch Flavor & Fragrance Co., Ltd., Guangzhou, China
Tamarind Extraction	Hangzhou Jiemin Flavor & Fragrance Co., Ltd., Hangzhou, China
Aglain Odorate Concrete	Hunan Jiali Flavor Co., Ltd., Yiyang, China
Turkish Oriental Tobacco Extraction	Guangzhou Huafang Tobacco Flavor Co., Ltd., Guangzhou, China
Tulip Concrete	Shanghai Qiyuan Flavor & Fragrance Co. Ltd., Shanghai, China
Fig Extraction	Guangzhou Huafang Tobacco Flavor Co., Ltd., Guangzhou, China
Smoked Plum Extraction	Shanghai Qiyuan Flavor & Fragrance Co. Ltd., Shanghai, China
Valerian Root Extraction	Guangzhou Huafang Tobacco Flavor Co., Ltd., Guangzhou, China
Tobacco Maillard Reactants	Guangzhou Huafang Tobacco Flavor Co., Ltd., Guangzhou, China
Yunnan Tobacco Extraction	Guangzhou Huafang Tobacco Flavor Co., Ltd., Guangzhou, China

Table S10. Detailed information of the reagents.

Reagents	Purity	Manufacturers
KH_2PO_4	ACS reagent,≥99.0%	Sigma-Aldrich (Shanghai) Trading Co.Ltd., Shanghai, China
TSP	99 atm% D	Sigma-Aldrich (Shanghai) Trading Co.Ltd., Shanghai, China
NaOD	40wt.%in D2O,99atm%D	Sigma-Aldrich (Shanghai) Trading Co.Ltd., Shanghai, China
DMSO-d_6	99.8 atm% D,	Ningbo Cuiying Chemical Technology Co., Ltd., Ningbo, China
D_2O	99.9 atm % D	Ningbo Cuiying Chemical Technology Co., Ltd., Ningbo, China
CD3OD	≥99.8 atm % D	Ningbo Cuiying Chemical Technology Co., Ltd., Ningbo, China
